# Possible role of ALDH1 and CD44 in lip carcinogenesis

**DOI:** 10.1590/1678-7757-2023-0227

**Published:** 2023-12-22

**Authors:** Rafael Carneiro ORTIZ, Gabriele Gomes GOIS, Camila Alves COSTA, Nádia Lago COSTA, Camila Oliveira RODINI

**Affiliations:** 1 Universidade de São Paulo Faculdade de Odontologia de Bauru Departamento de Ciências Biológicas Bauru SP Brasil Universidade de São Paulo , Faculdade de Odontologia de Bauru , Departamento de Ciências Biológicas , Bauru , SP , Brasil .; 2 Universidade Federal de Goiás Faculdade de Odontologia da Goiânia Centro de Pesquisa em Saúde Bucal Sistêmica da Goiás Goiânia GO Brasil Universidade Federal de Goiás , Faculdade de Odontologia da Goiânia , Centro de Pesquisa em Saúde Bucal Sistêmica da Goiás , Goiânia , GO , Brasil .; 3 Universidade Federal de Goiás Faculdade de Odontologia Departamento de Patologia Bucal, Estomatologia e Radiologia Goiânia GO Brasil Universidade Federal de Goiás , Faculdade de Odontologia , Departamento de Patologia Bucal, Estomatologia e Radiologia , Goiânia , GO , Brasil .

**Keywords:** Cancer stem cells, ALDH1, CD44, Actinic cheilitis, Lip squamous cell carcinoma

## Abstract

Lip squamous cell carcinoma (LSCC) accounts for 12% of all head and neck cancers. It is caused by chronic exposure to ultraviolet light solar radiation and related to previous actinic cheilitis (AC). This study aimed to investigate the immunostaining of the putative cancer stem cells (CSC) markers ALDH1 and CD44 in AC (n=30) and LSCC (n=20). ALDH1 positivity was found to be statistically higher in LSCC than in AC lesions (p=0.0045), whilst CD44 expression was statistically higher in AC than in LSCC lesions (p=0.0155). ALDH1+ cells in AC lesions were associated with specific clinical features: a younger age (<60 years old), the female gender, white skin, not smoking or consuming alcohol, and a fast evolution, and not associated with the chronic exposure to UV radiation (p<0.0001). CD44 positivity was associated with patients who were male, feoderm, smoked, consumed alcohol, underwent occupational exposure to UV-radiation, and demonstrated lesions with log-time evolution (p<0.0001). ALDH1 ^+^ cells were associated with mild dysplasia using a system from the World Health Organization (WHO), and with a low risk of malignant transformation, according to the binary system (p<0.0001). CD44+ cells were also associated with moderated dysplasia, according to the WHO system. In LSCC, ALDH1 ^+^ cells were positively associated with patients who were older (≥ 60 years old), smokers, and with those who consumed alcohol (p<0.0001). CD44 ^+^ cells in LSCC were associated with older (≥ 60 years old) patients as well, but also with female patients, white skin, non-smokers, and individuals who did not consume alcohol (p<0.0001), all of whom showed distinct patterns in pre- and malignant lesions of both markers. Additionally, in LSCC, both ALDH1 and CD44 staining were associated with smaller tumor sizes (T1/T2; p<0.0001). In summary, although both ALDH1 and CD44 were associated with the presence of dysplasia in AC lesions, the present findings suggest that ALDH1 and CD44 may be activated by different etiopathogenic pathways, predominantly in distinct steps of oral carcinogenesis. CD44 would thus be more significantly related to the potentially malignant lesion, while ALDH1 would be closely linked to malignancy.

## Introduction

Actinic cheilitis (AC) is a potentially malignant disorder (PMD) caused by the chronic exposure to ultraviolet (UV) radiation, which leads to genetic and epigenetic alterations in epithelial cells. ^1^ In approximately 10-20% of cases, AC undergoes malignant transformation into lip squamous cell carcinoma (LSCC), ^2^ which accounts for 25-30% of malignant neoplasms in the oral cavity. ^3^ Both AC and LSCC are primarily caused by UV exposure and specific skin phenotypes, and are more common in individuals with white skin. ^4^ Although LSCC generally has a better prognosis than other malignant tumors in the oral cavity, ^5^ the prognosis rate for this condition varies across global regions and depends on the presence of cervical lymph node metastasis, which can significantly worsen LSCC prognosis ^6^ and lead to a reduced overall survival rate. ^7^

While metastasis is known to be associated with a poor prognosis, the mechanism by which tumor cells detach from primary tumors and colonize different sites is not yet fully understood. Recent studies have shown that a small population of cancer cells called cancer stem cells (CSCs) is responsible for the development and spread of carcinoma cells. ^8^ This subpopulation is characterized by an indefinite self-renewal capacity and resistance to apoptosis, and leads to tumor progression and metastasis. ^9^ Several surface molecules have been identified as CSC markers, most of them related to the regulation of CD44 and ALDH1 molecules. ^10^ CD44 is a transmembrane glycoprotein receptor for hyaluronan. It is a widely recognized marker for CSCs and is notably associated with the progression of Head and Neck Squamous Cell Carcinoma (HNSCC). ^11^ Aldehyde dehydrogenase 1 (ALDH1) is a detoxifying enzyme involved in cell differentiation, cell detoxification, and drug resistance, and is also associated with a poor prognosis in cancer. ^12^

Recent studies have investigated the association of CD44 and ALDH1 expression with HNSCC regulation and progression, using *in vivo* and *in vitro* models. ^13^ It is important to note that another study conducted by our research group has already investigated the immunoexpression of ALDH1 and CD44 in the primary tumor of oral squamous cell carcinoma (OSCC) and in corresponding cervical metastatic lymph nodes. In this previous study, we performed a semi-quantitative analysis based on scores assigned according to the proportion and intensity of positive cells within the invasive front in the primary OSCC and in metastatic lymph nodes as a whole. Our findings demonstrated that the high expression of CD44 was a predictor of lymph node metastasis and that high ALDH1 immunostaining was associated with angiolymphatic invasion, showing that CD44 and ALDH1 play an important role in OSCC invasion and metastasis. ^14^

A similar study investigated the expression of CSC markers CD44, ALDH1, and p75NTR in UV-induced lesions, including AC and LSCC, using immunohistochemistry. The authors observed, continuously, that membrane immunostaining was positive for CD44, cytoplasmic immunostaining was positive for ALDH1, and both types of staining were positive for p75 in potentially malignant and malignant lip lesions. However, they did not evaluate these markers according to clinicopathological data collected during cancer progression. ^15^ CSC expression has been confirmed to occur in OSSC ^14 , 15^ , but this conclusion was taken using simple markers, not entirely specific ones—which means that the combination of a set of markers is needed in order to improve the specificity of the recognition and isolation of CSCs. ^16^ There is a shortage of reports assessing the presence and role of CSCs in AC and LSCC, as well as their potential clinical significance. ^17^ This is the first immunohistochemical study designed to evaluate the expression of the CSC markers CD44 and ALDH1 in AC and LSCC lesions, and to correlate them with patients’ clinicopathological data.

## Methodology

### Samples

Archived formalin-fixed paraffin-embedded tissue samples of 30 patients with AC and 20 patients with LSCC were obtained from the Oral Pathology Laboratory, School of Dentistry, Federal University of Goiás (FO-UFG), between January 2003 and December 2011. Samples of patients with systemic diseases or who had undergone preoperative treatments such as radiotherapy or chemotherapy were excluded from this study. Table 1 lists the clinical and pathological features of the samples. This study was approved by the local Research Ethics Committee of Bauru School of Dentistry (protocol 50695815.2.0000.54.17), and all participants signed an informed consent form. The data supporting the findings of this study are available from the corresponding author upon reasonable request.

**Table 1:** Clinicopathological features of patients with AC and LSCC

Clinical features		Cases	
		**AC**	**LSCC**
		**n (%)**	**n (%)**
Gender	Female	6 (20%)	5 (25%)
	Male	24 (80%)	15 (75%)
Race	White skin	24 (80%)	11 (55%)
	Feoderm	5 (16%)	6 (30%)
	Melanoderma	0	0
	Albino	1 (3%)	0
	NI	1 (3%)	3 (15%)
Smoker	Yes	4 (13%)	13 (65%)
	No	11 (36%)	5 (25%)
	NI	15 (50%)	2 (10%)
Alcohol consumption	Yes	5 (16%)	4 (20%)
	No	9 (30%)	7 (35%)
	NI	16 (53%)	9 (45%)
Age	>40	2 (6%)	1 (5%)
	41-50	6 (20%)	2 (10%)
	51-60	7 (23%)	3 (15%)
	61-70	11 (36%)	5 (25%)
	71-80	4 (13%)	8 (40%)
	81-90	0	1 (5%)
Dysplasia	No	9 (30%)	NA
	Mild	13 (43%)	NA
	Moderate	5 (16%)	NA
	Severe	3 (10%)	NA
T category	1	NA	9 (45%)
	2	NA	7 (35%)
	3	NA	2 (10%)
	4	NA	2 (10%)
Metastasis	Yes	NA	1 (5%)
	No	NA	19 (95%)
Binary system	Low	24 (80%)	NA
	High	6 (20%)	NA

### Histopathologic analysis

**Figure 1:**
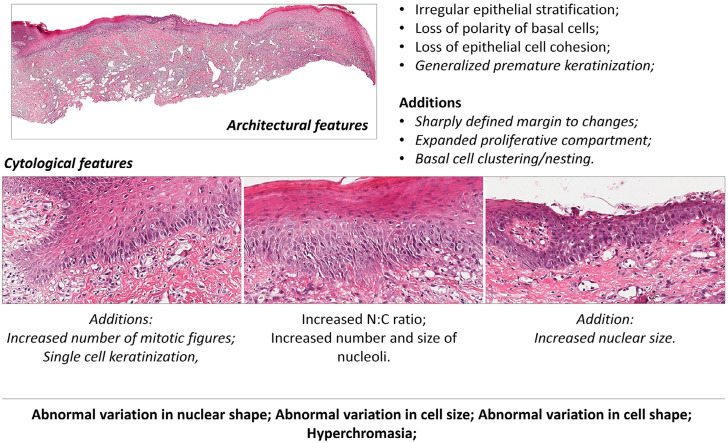
A representative case demonstrating some architectural cytological features of reclassified AC dysplasia

Hematoxylin and eosin (H&E) slides from 5-μm sections of AC and LSCC underwent a rigorous evaluation conducted by two experienced oral pathologists. The primary objectives of this evaluation were to confirm the AC and LSCC diagnoses, ascertain the presence or absence of epithelial dysplasia in samples displaying AC, and establish the histological grade of LSCCs. This assessment was carried out in accordance with the guidelines outlined in the WHO criteria formulated by Barnes, et al. ^18^ (2005) and updated in 2022 by Muller and Tilakaratne ^19^ (2022), which included a comprehensive expansion of architectural and cytological features specific to oral epithelial dysplasia. Notably, these criteria included distinctions related to keratinization, encompassing generalized premature keratinization in architectural features characterized by cells in the lower spinous layer with prominent eosinophilic cytoplasm, as well as single cell keratinization in cytological features. ^19^ Figure 1 shows a representative case with some architectural cytological features of AC dysplasia.

It is essential to acknowledge that the use of the three-tiered oral epithelium dysplasia grading system can lead to an oversimplification of the intricate nature of dysplasia. Consequently, in our study, AC epithelial dysplasia was also analysed by the binary grading system proposed by Kujan, et al. ^20^ (2006), as high or low risk of malignant transformation. Additionally, the presence of solar elastosis within the connective tissue of both AC and LSCC specimens was carefully assessed as part of this comprehensive methodology.

### Immunohistochemistry

The immunoexpression of ALDH1 and CD44 in AC and LSCC tissue samples was analyzed by immunohistochemistry. The 3-μm sections from these tissue specimens were dewaxed, rehydrated, and subjected to antigen retrieval in Steamer (NT-0334C, FK) using citrate-buffer solution pH 6,0 (Sigma Aldrich), at 95°C for 30 min. To block endogenous peroxidase activity, the sections were incubated in 3% hydrogen peroxide for 10 minutes, and to suppress the nonspecific binding of subsequent reagents, they were incubated with Protein Block Serum-Free (Dako, Carpinteria, CA) for 15 minutes at room temperature (RT). The sections were then incubated for one hour at RT with the following primary antibodies: mouse anti-human ALDH1 (clone 44, cat# 611195, 1:700, BD Biosciences, NJ, USA) or mouse anti-human CD44 (clone 156-3C11, cat#3570, 1:700, Cell Signaling, MA, USA) monoclonal antibodies. They were subsequently incubated with the secondary biotin-free peroxidase-based ADVANCE™ kit (K4068, Dako) for one hour at RT, then with the 3´3-diaminobenzidine (DAB) substrate-chromogen system (Dako) for 3 minutes, and lastly subjected to counterstaining with Mayer’s hematoxylin. Negative controls were also treated with the procedures described above but omitting the primary antibody from the reaction sequence. Breast cancer tissue specimens and lymphocytes from cervical lymph nodes were used as positive controls for ALDH1 and CD44, respectively.

### Immunostaining quantification

CD44 and ALDH1 immunoreactivity was quantitatively and qualitatively assessed within tumor cells by two examiners who were blinded to the clinicopathological parameters. For the quantification, images from the entire tissue sections (about 20 microscopic fields) were captured and counted at a final magnification of x400 (using the AxionVision Software, Carl Zeiss). It is important to note that the number of total and immunopositive cells were counted within the entire epithelium of AC lesions, without distinguishing between layers, and at the center and invasive front (the deepest field with cancer cell infiltration) in specimens with LSCC. To account for the notable size differences between AC and LSCC lesions, we adopted a proportional approach, calculating the percentages of positive cells relative to the total cell count. This method facilitated the comparison between the CSC marker expression levels of these distinct lesion types. Additionally, to analyze clinicopathological variables, we categorized positive tumor cell counts into low and high CSC immunostaining groups, as these parameters were assessed in a binary manner.

Due to the variability in the range of cut-off values among different CSC markers, to set a standardized approach, we calculated the mean percentage of positive cells across all lesions. This mean value was subsequently used to divide the results into two categories: low and high positivity, both for both ALDH1 (< 5% of positive cells and ≥ 5% of positive cells, respectively) and CD44 (< 78% of positive cells and ≥ 78% of positive cells, respectively). This method ensured consistency and comparability in the categorization of different CSC marker expression levels. ^11^

### Statistics

The clinicopathological characteristics of patients and protein immunoexpression were analyzed. The total number of cancer cells that were positive for ALDH1 and CD44 immunostaining among AC lesions and tumor sites were compared with the Unpaired T test followed by the Mann-Whitney test. When simultaneous immunostaining evaluations were attainable for both markers, which was the case of a cohort comprising 26 AC and 10 LSCC specimens, statistical analyses were conducted using the Pearson Correlation Test. The Chi-square Test was used to assess the correlation between clinicopathological findings and protein immunostaining. Kaplan–Meier estimators were used to compare the probability of survival of the different groups. Statistical analyses were performed using the GraphPad Prism 7 (GraphPad software, Inc., CA, USA), and P<0.05 was considered statistically significant.

## Results

### Sample characterization

**Figure 2:**
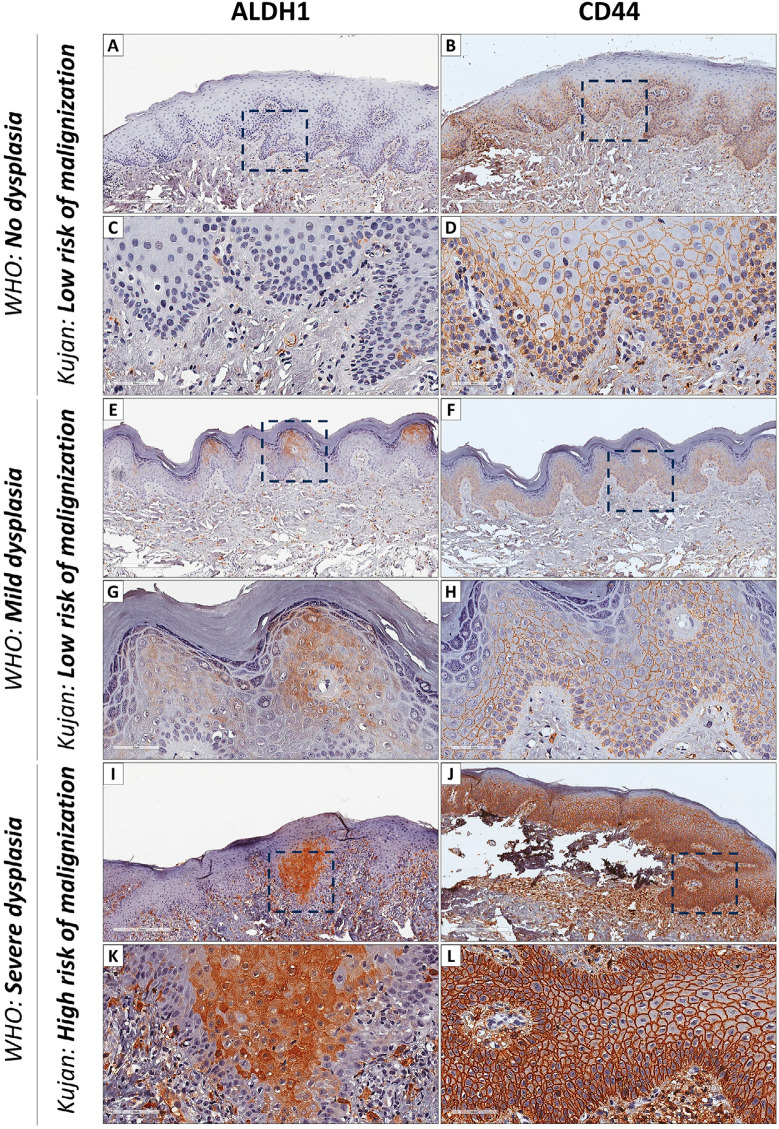
Immunohistochemical Analysis of AC Lesions with Varying Dysplastic Changes. (A) Nondysplastic AC lesions classified as having a low risk of malignant transformation show no ALDH1 expression. (B) In the same low-risk AC lesions, a uniform granular CD44 staining pattern can be seen along the membranes of epithelial cells within the basal and spinous layers. (C) High view of the low-risk AC lesion confirming the absence of ALDH1 expression. (D) CD44-positive lymphocytes primarily localized in the subepithelial region of the low-risk AC lesions. (E) AC lesions demonstrating mild dysplasia exhibit focal areas with ALDH1-positive epithelial cells within the spinous and granular layers of the epithelium. (F) CD44 staining pattern in mild dysplasia AC lesions closely resembles that of nondysplastic AC lesions, observed in the membranes of epithelial cells within the basal and spinous layers. (G) High view of the mild dysplasia AC lesion showing ALDH1-positive epithelial cells in focal areas. (H) CD44 staining in mild dysplasia AC lesions continues to resemble that of nondysplastic AC lesions. (I) AC lesions categorized as having a high risk of malignancy exhibit ALDH1-positive epithelial cells concentrated in focal areas, with robust cytoplasmic staining in both normal and altered cells. (J) In high-risk AC lesions, CD44 shows a strong and uniform positivity throughout the entire population of epithelial cells, except in parakeratinized layers, where CD44 expression is notably absent. (K) High view of the high-risk AC lesion showing ALDH1-positive epithelial cells in focal areas. Perilesional macrophages exhibit a pronounced ALDH1-positive phenotype. (L) The CD44 staining in high-risk AC lesions remains strong, except in the parakeratinized layers, where it is absent

Most of the patients with AC were male (n=24; 80%), white skin (n=24; 80%), were aged between 61 and 70 years (n=11; 36%), did not smoke (n=11; 44%), and did not drink (n=11; 36%). According to the WHO grading system for [30], most of the lesions presented microscopic features of dysplasia (n=21; 70%), either mild (n=13; 43%), moderate (n=5; 16%), or severe (n=3; 10%)—this was confirmed by the routine H&E staining ( Figure 1 ). Most AC lesions were classified as having a low risk of malignant transformation (n=24; 80%) (Kujan, et al. ^18^ (2006). The findings indicate that, after a comprehensive five-year follow-up period, 30% (9 out of 30) of individuals diagnosed with AC remained under surveillance, but no evidence indicated that their lesions were progressing toward malignant transformation into LSCC. In total, three (33.33%) of these patients exhibited no discernible dysplasia, three others (33.33%) displayed mild dysplasia, and the remaining 3 (33.33%) had moderate dysplasia ( Figure 2 ).

**Figure 3:**
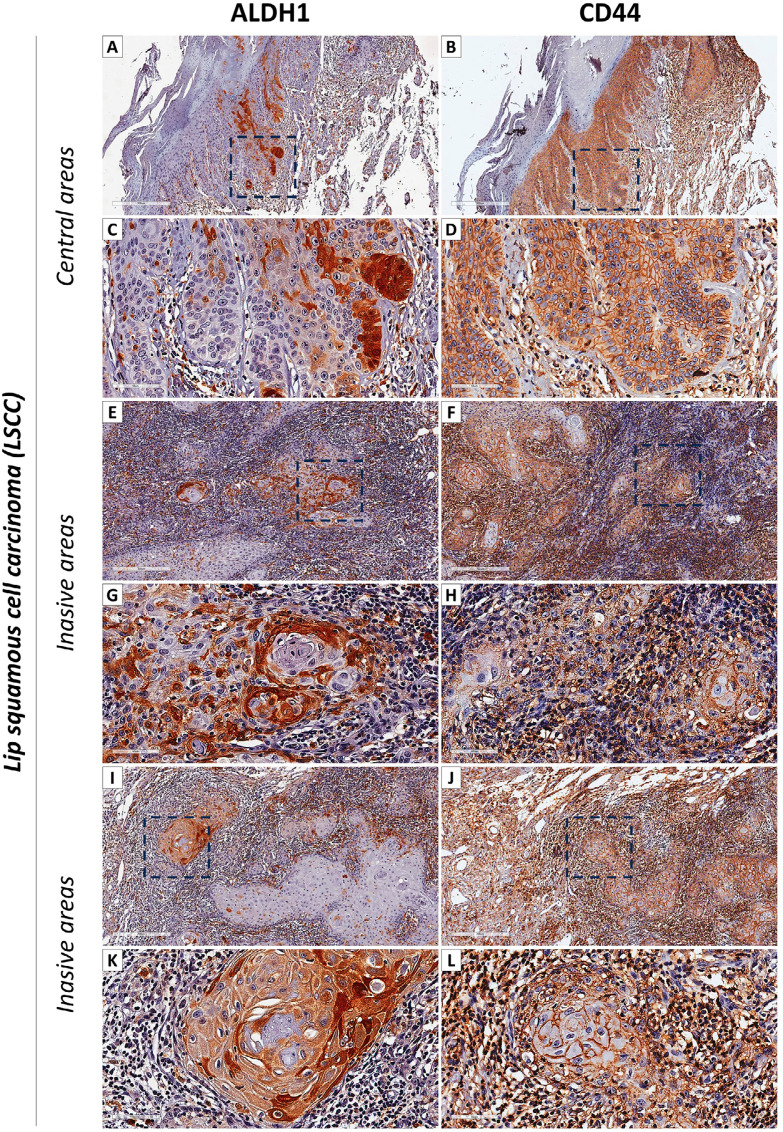
Immunohistochemical Analysis of LSCC Tumor Cells. (A) Within the central regions of LSCC lesions, predominantly ALDH1-positive tumor cells form niches extending from the basal layers to the superficial ones. (B) In these central regions, CD44 staining closely resembles that observed in AC dysplastic lesions. (C) High view of the central regions of LSCC lesions, highlighting the ALDH1-positive tumor cells extending from the basal layers to the superficial ones. (D) CD44 staining in the central regions continues to resemble that observed in AC dysplastic lesions. (E) In the invasive areas of LSCC, a distinctive staining pattern emerges, with mild staining for ALDH1. (F) In these invasive areas, there is strong staining for CD44, particularly within the core regions of tumor islands. (G) In the more invasive tumor regions, there is a notable shift in the staining profile, with invasive tumor cells exhibiting strong ALDH1 positivity. (H) Also in the more invasive tumor regions, there is a decrease in CD44 positivity. (I) High view of the invasive areas of LSCC, emphasizing the mild staining for ALDH1. (J) In these invasive areas, strong staining for CD44 is particularly evident within the core regions of tumor islands. (K) A close-up view of the more invasive tumor regions, showing invasive tumor cells with strong ALDH1 positivity. (L) In these more invasive tumor regions, CD44 positivity decreases as compared to less invasive areas

Most patients with LSCC were male (n=15; 75%), white skin (n=11; 55%), were aged 71 to 80 years (n-=8; 40%), and smoked (n=13; 65%). According to the WHO grading system, the majority of the lesions were small (T1) (n=9; 45%) and did not generate metastases (n=19; 95%). In addition, all lesions were histologically well-differentiated tumors (Figures 3G and J). Table 1 details all the clinical features measured.

### ALDH1 and CD44 immunostaining

Qualitatively, CSC markers exhibited distinct immunolabeling patterns in terms of distribution and intensity, both in AC and LSCC. ALDH1 staining was focal and restricted to the cytoplasm, while CD44 staining was widely expressed in the membrane of normal, dysplastic and tumor epithelial cells. Epithelial cells from AC lesions were predominantly negative for ALDH1 (75%) and mostly positive for CD44 (90%).

In general, ALDH1 was not identified within the nondysplastic lip transitional region of AC lesions classified as having a low risk of malignant transformation (Figures 2A and C). Furthermore, a uniform granular CD44 staining pattern could be seen along the membranes of epithelial cells within the basal and spinous layers (Figures 2B and E). Figure 2D shows the identified CD44-positive lymphocytes, which were primarily localized in the subepithelial region. In contrast, AC lesions with mild dysplasia exhibited focal areas with ALDH1-positive epithelial cells within the spinous and granular layers of the epithelium (Figures 2E and G). Additionally, Figure 2E highlights the presence of ALDH1-positive macrophages within the solar elastosis. Remarkably, the CD44 staining pattern observed in the membranes of epithelial cells within the basal and spinous layers closely resembles that of nondysplastic AC lesions (Figures 2F and H). Conversely, in AC lesions categorized as having a high risk of malignant transformation, ALDH1-positive epithelial cells were concentrated in focal areas, with robust cytoplasmic staining in both normal and altered cells (Figures 2I and K). Moreover, perilesional macrophages exhibited an evidently ALDH1-positive phenotype (Figure 2K). A strong and uniform positivity for CD44 was identified throughout the entire population of epithelial cells, except in parakeratinized layers, where CD44 expression was notably absent (Figures 2J and L).

Most LSCC tumor cells did not express ALDH1 (94.5%) but did express CD44 (75%) ( Figure 3 ). A histological examination of all LSCC lesions consistently revealed well-differentiated tumors with minimal histological variations across biopsies ( Figure 3 ). Interestingly, within the central regions of LSCC lesions, we observed preferentially ALDH1-positive tumor cell niches extending from the basal layers to the superficial ones (Figures 3A and C). Furthermore, CD44 staining in these central regions closely resembled the one observed in AC dysplastic lesions (Figures 3B and D). In the invasive areas of LSCC, a distinctive staining pattern emerged. These areas exhibited mild staining for ALDH1 (Figures 3E and I) and strong staining for CD44 (Figures 3F and J), particularly within the core regions of tumor islands. Intriguingly, in the more invasive tumor regions, there was a notable shift in the staining profile: invasive tumor cells exhibited strong ALDH1 positivity (Figures 3G and K) and decreased CD44 positivity (Figures 3H and L).

**Figure 4:**
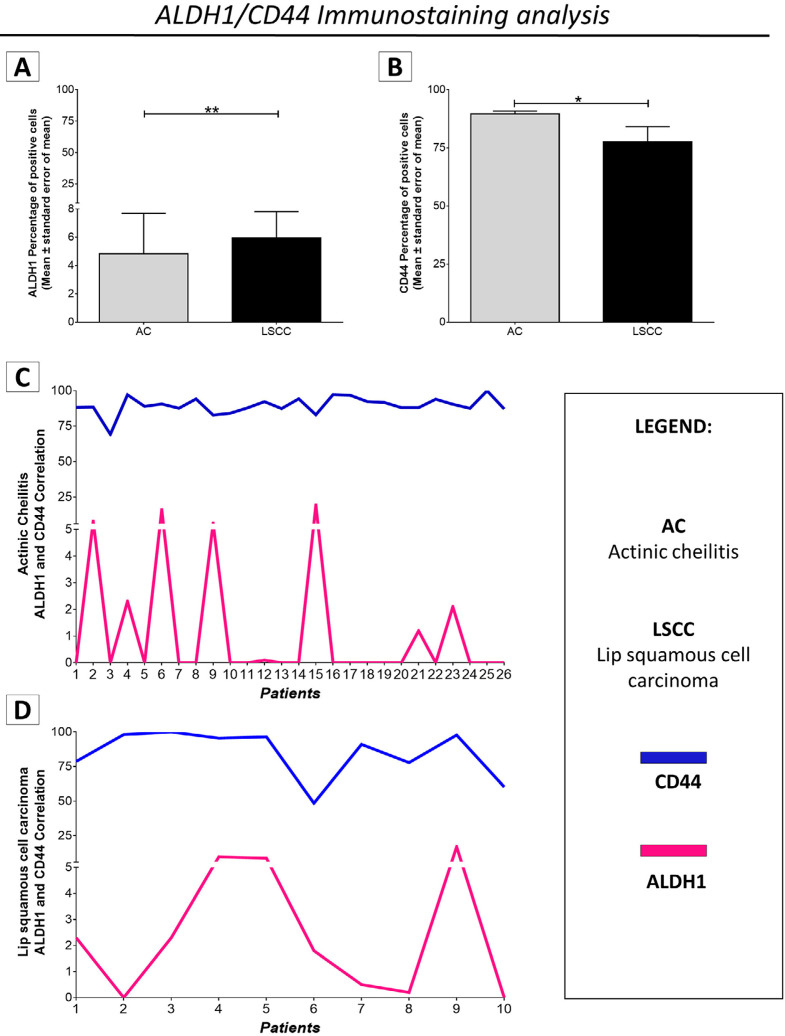
Comparison between the immunoexpression level of CSC markers in AC and LSCC. (A) ALDH1+ cell concentrations were statistically higher in LSCC (5.944 ± 1.867) than in AC (4,826 ± 2.862) (p= 0.0045). (B) CD44+ cell concentrations were statistically higher in AC (89.6 ± 1.197) than in LSCC (77.56 ± 6.575) (p= 0.0155). (C) CD44 and ALDH were negatively correlated in AC (r= -0.191; p=0.350), and (D) positively correlated in LSCC (r=0.438; p=0.206), although without statistically significance. A-B: Mann-Whitney Test; C-D: Person correlation

Quantitatively, the comparison between the immunoexpression level of CSC markers in AC and LSCC revealed that LSCCs had a statistically significant percentage of ALDH1 ^+^ cells, higher (mean of positive cells ± standard error of mean) (5.944±1.867) than the one exhibited by AC lesions (4,826±2.862) (p=0.0045; *Mann-Whitney Test* ; Figure 4A). The ratio for CD44 protein markers was reversed: AC lesions were found to have a higher statistically significant percentage of CD44 ^+^ cells (mean of positive cells ± standard error of mean) (89.6±1.197) than LSCCs (77.56±6.575) (p=0.0155; *Mann-Whitney Test* ; Figure 4B). When concurrent immunostaining assessments were feasible for both markers, which was the case of a cohort with 26 AC and 10 LSCC samples, the Pearson correlation test did not indicate statistically significant associations. However, it should be noted that CD44 and ALDH did exhibit a modest negative correlation within the AC cohort (r= -0.191; p=0.350; Figure 4C). Conversely, in LSCC lesions, a discernible positive correlation between CD44 and ALDH was observed (r=0.438; p=0.206; Figures 4D).

The assessment of associations of ALDH1 and CD44 immunopositivity with AC or LSCC data revealed that the clinical patterns for each marker were very diverse within both lesions—Tables 2 and 3 summarize the data that led to this finding. In AC lesions ( Table 2 ), ALDH1 ^+^ cells were statistically associated with patients who were aged <60 years (p<0.0001), were female (p<0.0001), white skin (p<0.0001), did not smoke (p<0.0001), did not consume alcohol (p<0.0001), were not chronically exposed to sunlight (p<0.0001), and had rapidly evolving lesions (up to 5 years) (p<0.0001). CD44 ^+^ cells, on the other hand, were statistically significantly associated with patients who were male (p<0.0001), feoderm (p<0.0001), consumed alcohol (p<0.0001), were chronically exposed to sunlight (p<0.0001), and had slow developing (> 5 years) lesions (p<0.0001). Following the WHO grading system, it was found that ALDH1 ^+^ cells were statistically significantly associated with mild dysplasia (p<0.0001) and positively linked to a low risk of malignant transformation (p<0.0001), while CD44 ^+^ cells were statistically significantly associated with moderated dysplasia (p<0.0001), but not linked to a risk of malignant transformation (p>0.05).

**Table 2:** Assessment of associations of ALDH1 and CD44 immunopositivity with the clinicopathological features of AC

	ALDH1						CD44				
Characteristics	Category	Negative	(%)	Positive	(%)	P	Negative	(%)	Positive	(%)	P
Age	<60	38190	95.1	1975	4.9	<0.0001*	4067	10.6	34418	89.4	0.0523
	≥60	27588	97.1	836	2.9		2989	10.1	26576	89.9	
Gender	Female	12319	89	1515	11	<0.0001*	1634	12.8	11113	87.2	<0.0001*
	Male	53459	97.6	1296	2.4		5422	9.8	49881	90.2	
Ethnic group	White skin	56385	95.3	2790	4.7	<0.0001*	6131	10.5	52239	89.5	<0.0001*
	Feoderm	9025	99.8	21	0.2		882	9.4	8527	90.6	
	Albino	368	100	0	0		43	15.9	228	84.1	
Smoker	Yes	10468	100	0	0	<0.0001*	3384	10.1	30055	89.9	0.1453
	No	26283	94.7	1457	5.3		1117	10.6	9405	89.4	
	N.A.	29027	95.5	1354	4.5		2667	11.4	20650	88.6	
Alcohol consumption	Yes	17315	99.3	123	0.7	<0.0001*	1772	10.1	15815	89.9	<0.0001*
	No	16750	92.6	1334	7.4		2940	11.9	21797	88.1	
	N.A.	31713	95.9	1354	4.1		2344	9.1	23382	90.9	
Evolution	≤5	24016	92.02	2082	8	<0.0001*	2784	13.3	18208	86.7	<0.0001*
	>5	22100	97.49	569	2.5		1852	7.2	23968	92.8	
	NI	19662	99.2	160	0.8		2420	9.9	21947	90.1	
Occupacional exposure to sunlight	Yes	34311	95.9	1456	4.1	<0.0001*	3512	10.4	30332	89.6	<0.0001*
	No	12575	90.4	1334	9.6		1904	14.6	11173	85.4	
	N.A.	18892	99.9	21	0.1		1640	7.8	19489	92.2	
Dysplasia presence or absence	Absence	27800	98.5	637	1.5	<0.0001*	1967	10	17661	90	0.0584
	Presence	37978	78.4	2174	4.5		5089	10.5	43333	89.5	
Dysplasia WHO grade	No	27800	98.5	637	1.5	<0.0001*	1967	10	17661	90	<0.0001*
	Mild	22875	92.8	1806	7.2		3197	11.1	25528	88.9	
	Moderate	9489	96.3	368	3.7		1235	7.8	14563	92.2	
	Severe	5614	100	0	0		657	16.9	3242	83.1	
Dysplasia Binary sistem	Low risk	53737	95.7	2443	4.3	<0.0001*	5328	10.3	46348	89.7	0.3775
	High risk	12041	97	368	3		1728	10.6	14646	89.4	

In LSCC ( Table 3 ), ALDH1 ^+^ cells were positively associated with patients who were aged ≥ 60 years old (p<0.0001), smoked (p<0.0001), consumed alcohol (p<0.0001), and had tumor sizes T1/T2 (p<0.0001). CD44 ^+^ cells were associated with patients who were female (p<0.0001), aged 60 years or more (p<0.0001), white skin (p<0.0001), did not smoke (p<0.0001) or consume alcohol (p<0.0001), and had tumor sizes T1/T2 (p<0.0001).

**Table 3:** Assessment of associations of ALDH1 and CD44 immunopositivity with the clinicopathological features of LSCC

	ALDH1						CD44				
Characteristics	Category	Negative	(%)	Positive	(%)	P	Negative	(%)	Positive	(%)	P
Age	<60	23728	99%	288	1%	< 0.0001*	5332	31%	11795	69%	< 0.0001*
	≥60	64833	93%	4905	7%		4144	19%	17911	81%	
Gender	Female	33727	95%	1921	5%	0.1188	1546	21%	5725	79%	< 0.0001*
	Male	54834	94%	3272	6%		7930	25%	23981	75%	
Ethnic group	White skin	56200	95%	2900	5%	0.1089	2697	16%	14456	84%	< 0.0001*
	Feoderm	22693	95%	1239	5%		6725	34%	12831	66%	
	NI	9668	90%	1054	10%		54	2%	2419	98%	
Smoker	Yes	46953	96%	2100	4%	< 0.0001*	8904	31%	19981	69%	< 0.0001*
	No	6770	98%	159	2%		2	0%	4034	100%	
	N.A.	34838	92%	2934	8%		570	9%	5691	91%	
Alcohol consumption	Yes	12586	90%	1405	10%	< 0.0001*	2433	30%	5655	70%	< 0.0001*
	No	30826	97%	1072	3%		4661	22%	16324	78%	
	N.A.	45149	94%	2716	6%		2382	24%	7727	76%	
T category	T1/T2	78835	94%	5004	6%	< 0.0001*	7919	22%	28640	78%	< 0.0001*
	T3/T4	9726	98%	189	2%		1557	59%	1066	41%	
	NI	19662	99.2	160	0.8		2420	9.9	21947	90.1	

**Figure 5:**
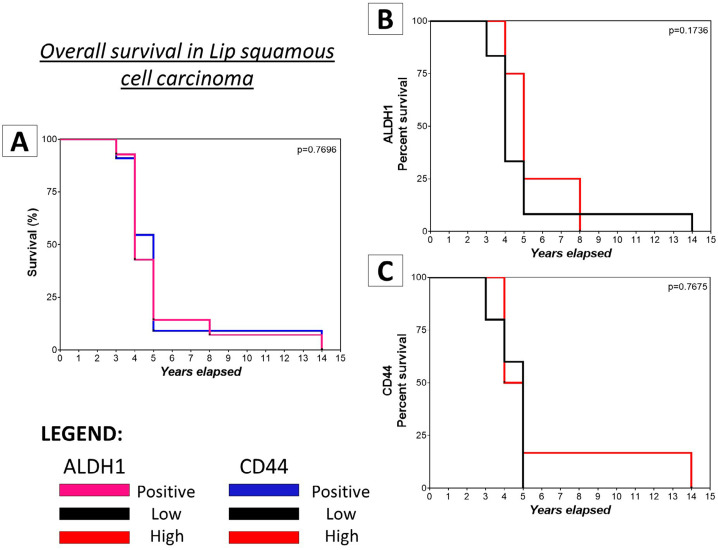
Overall survival of patients with LSCC according to ALDH1 and CD44 immunostaining. (A) Overall survival of patients with LSCC. (B) ALDH1high patients had an overall survival rate > 5 years (average of 8 years; p = 0.1736). (C) CD44low patients had an overall survival rate ≤ 5 years (p = 0.7675). However, none of these values demonstrated statistical significance

Lastly, the dichotomized immunopositivity levels (high and low) of each marker were assessed in relation to overall survival. Although no significant correlation was observed between ALDH1 ^+^ or CD44 ^+^ immunoexpression levels and patients’ overall probability of survival, it was found that patients with LSCC who were ALDH1 ^high^ had an overall survival rate > 5 years (average of 8 years; p=0.1736) while patients who were CD44 ^low^ had an overall survival rate ≤ 5 years (p=0.7675) ( Figure 5 ).

## Discussion

This study has shown that ALDH1 ^+^ cells were more prevalent in LSCC than in AC sites, unlike CD44, which is more prevalent in AC lesions. According to clinical and social features, ALDH1 was associated with fast AC development in patients who were younger, female, white skin, did not consume alcohol or tobacco, and were not chronically exposed to UV radiation. Conversely, CD44 was associated with slow AC development in patients who were male, feoderm, consumed alcohol, and were chronically exposed to UV radiation. Furthermore, in AC lesions, ALDH1 was associated with mild dysplasia, according to the WHO grading system, and with a low risk of malignant transformation, according to the binary grading system, while CD44 was associated with moderate dysplasia, also according to the WHO system. In LSCC, however, ALDH1 was associated with patients who were older, smoked, and consumed alcohol, while CD44 was associated with patients who were older, female, white skin, smoked, and consumed alcohol. Additionally, both markers were associated with small (T1/T2) LSCC tumors, but showed distinct patterns concerning CSC markers according to pre- and malignant lesions.

Our study reports the presence of focal and rarely cytoplasmic ALDH1 predominantly in the squamous epithelial of non- and dysplastic AC lesions. In LSCC, ALDH1-positive tumor cells were focal and mainly immunoexpressed in the central regions of tumor nests. These cells had statistically higher concentrations in LSCC lesions than in AC lesions, which indicates that they play a key role in tumor establishment and could be associated with the progression of AC to LSCC. ALDH1 expression has been reported to occur in various types of human carcinomas, including OSCC. ^21^ Recently, Thankappan, et al. ^22^ (2022) demonstrated that 71.43% of oral epithelial dysplasia cases showed positive ALDH1 expression, with higher mean scores in moderate and severe dysplasia. ^23^ These results contradict our findings, which indicated that ALDH11 ^+^ cells were associated with mild dysplasia and a low risk of malignant transformation. Although it is known that about 10-20% of AC lesions can progress to LSCC, ^24^ our findings substantiate the idea that ALDH1 could be directly involved in the malignant transformation of AC into LSCC.

In regard to CD44, positive staining was observed in the basal and spinous layers of the dysplastic epithelium of AC, and homogenous staining was found in LSCC. Overall, this staining was statistically higher in AC than in LSCC. CD44 exhibited a near-ubiquitous presence within the cellular strata of the oral epithelium—its expression levels varied primarily in intensity, which was contingent upon specific cell layers or individual cells. ^25^ Moreover, CSCs were initially characterized *in vitro* , with a focus on CD44 expression as delineated through flow cytometry, which resulted in the identification of CD44 ^bright^ and CD44 ^dim^ subpopulations. These studies compellingly demonstrated that the CD44 ^bright^ population, unlike the CD44 ^dim^ one, can generate a heterogeneous tumor and exhibit a self-regenerative potential when transplanted into immunocompromised murine models. ^10^ After these pivotal findings were made, numerous other *in vitro* investigations indicated that CD44 ^bright^ cells are the major CSC subpopulation in OSCC. ^26 - 27^ Nonetheless, as articulated by Sadasivam, et al. ^28^ (2020), questions about the clinical relevance of CSC quantification still arise, especially when specialists attempt to predict risks of malignant transformation and of key clinical parameters such as tumor stage, invasiveness, and metastatic potential, highlighting a conspicuous correlation between the elevated expression of CSC biomarkers, including CD44, and the adverse prognostic outcomes in Head and Neck cancers and OSCC. ^28^ In this study, we also observed that CD44 ^+^ cells were associated with moderate dysplasia, which contradicts the results obtained by Thankappan, et al. ^22^ (2022), who found similar levels of CD44 expression in all stages of oral dysplasia, regardless of its level. ^22^ Umeda, et al. ^29^ (2016), on the other hand, demonstrated that a decrease in CD44 expression may play a crucial role in promoting second tumor sites by reducing or eliminating the peritumoral stroma binding ability, ^29^ which indicates that CD44 may play different roles in the temporal and specific features of oral carcinogenesis.

We also evaluated clinicopathological findings concerning the expression of lesions and CSC biomarkers in order to understand how these molecules could be involved in lip tumorigenesis. An unexpected clinical profile of AC patients was associated with ALDH1 immunoexpression: most patients with this trait were younger (<60 years old), female white skin, did not smoke, did not consume alcohol, had rapidly evolving (≤5 years) lesions, and were not chronically exposed to UV radiation. On the other hand, a more predictable clinical profile was observed in patients with LSCC who exhibited ALDH1 ^+^ cells: these individuals were older (≥ 60 years old), male, smoked, consumed alcohol, and had small tumors (T1/T2). The transformation of normal lip tissue mucosa into LSCC usually occurs gradually. It involves changes in cellular and tissue architecture, which can be clinically visible and categorized as PMD, similarly to other potentially malignant oral disorders. ^30^ This multistep process can provide great opportunities for early cancer detection; however, few studies have investigated the pathogenesis involved in the transformation of AC into LSCC.

We found that ALDH1 ^+^ cells were significantly associated with mild dysplasia and positively related to a low risk of malignant transformation in AC. Corroborating our findings, a recent report on oral PMD demonstrated a significant association of ALDH1 with dysplasia, regardless of their graduation. ^31^ Several other studies also demonstrated the prognostic value of CSCs markers, including ALDH1, in premalignant adenomatous polyps of colon and precancerous gastric lesions, ^32 , 33^ especially in the fast regulation of apoptosis resistance, self-renewal, and differentiation. ^34^ Our results also demonstrated that ALDH1 immunopositivity was higher in AC lesions than in LSCC. A similar observation was made by Visus, et al. ^35^ (2007), who used ALDH1 as a distinguishing marker for PMD and malignant lesions, ^32^ corroborating the idea that ALDH1 expression changes depending on the pathological variance of pre- and malignant lesions, and could contribute to the fast development and progression of cancer. Several reports have demonstrated that the chronic exposure to UV radiation and the consumption of alcohol and tobacco are carcinogenic risk factors for HNSCC, especially in older patients, due to the longer time of exposure. ^35^

Although our findings are in accordance with the abovementioned etiological factors regarding patients with LSCC, we observed that patients with AC had a different clinical profile. Nevertheless, there are still questions about the role of these etiological factors in people who are not exposed to these factors or undergo a short time exposure to them. ^23^ The ALDH1 enzyme is involved in the oxidation of acetaldehyde to acetate and presents various isoforms with several and novel roles in the development of head and neck cancer. ^36^ Zhou, et al. ^37^ (2020) demonstrated that the nitrosamine 4-(methylnitrosamino)-1-(3-pyridyl)-1-butanone (NNK) found in tobacco and exposed by smoking is the major responsible for carcinogenesis, as it upregulates the expression levels of ALDH1A1. These findings provide new insights on the role of CSCs in the precancer stage of malignant progression, demonstrating that ALDH1 can be associated with the presence of dysplasia, an important feature that is linked to cancer transformation and is independent of chronic UV radiation exposition.

It is commonly reported that CD44 plays several roles in head and neck carcinogenesis. ^10 , 11 , 14^ Interestingly, in our study, CD44 positivity was associated with patients who were male, feoderm, smoked, consumed alcohol consumer, and were occupationally exposed to UV-radiation. It was also associated with moderated dysplasia (WHO grade) and a log-time evolution of lesions. In LSCC, CD44 immunopositivity was associated with patients who were older (≥ 60 years old), male, white skin, did not smoke, and did not consume alcohol. As CD44 evidently has different roles across the stages of cancer development, it may be upregulated or downregulated by tumor environment cells, providing new information on the involvement of CSCs in the malignant transformation of oral cancer. These findings are consistent with the observations made by Mirhashemi, et al. ^38^ (2020), who concluded that an accurate examination of oral dysplastic lesions is important to predict and ultimately avoid OSCC transformation, since the majority of their sample displayed strong positivity for CD44.

Moreover, it is essential to highlight the significance of immune evasion mechanisms in malignant transformation across various diseases, including the role played by the PD-1/PD-L1 (programmed cell death protein 1/programmed death-ligand 1) axis. A recent investigation has shed light on the correlation between PD-L1 positivity within the membrane and the cytoplasm of dysplastic epithelial cells in AC and tumor cells in LSCC. This study revealed distinct associations between PD-L1 expression and an imbalance in the CD4+ and CD8+ lymphocyte populations, indicating a potential avenue for immune response evasion within oral lesions and thus highlighting the relevance of perilesional inflammatory infiltrates to influencing the prospects of malignant transformation. ^37^

It is important to point out that the CSC population can vary and express distinct markers depending on the stage of carcinogenesis. This variability suggests that a multi-marker panel of the CSC population in PMD, validated by multicenter prospective studies, could be more effective in predicting carcinogenesis. ^39^ Additionally, it is speculated that CSCs may go through a potentially malignant stage during the evolution of carcinomas, which would mean that CSCs and pre-CSCs in OPMD have characteristics of both CSCs and normal stem cells. ^40^

This study does have certain limitations, which should be acknowledged. One limitation pertains to the relatively modest sample size, which did not encompass a comprehensive representation of all types of dysplasia in AC and the various histological grades of LSCC. Future studies should expand this sample size to encompass a broader spectrum of cases. Additionally, the selection criteria for corresponding cases were somewhat restrictive, and the inclusion of a broader array of CSC markers could have enhanced the comprehensiveness of this study. However, it is noteworthy that despite these limitations, this study has distinctive strengths. Studies on CSC markers in LSCC and OPMD are very scarce in literature and this work adds valuable information to the field. Notably, the study reveals discernible patterns in the expression of two key CSC markers, ALDH1 and CD44, in both potentially malignant and malignant lesions. These markers appear to be activated through disparate etiopathogenic pathways, predominantly at different stages of oral carcinogenesis. This means that CD44 may hold greater significance in potentially malignant lesions, which reinforces the need for its early validation for its utility in detection. Conversely, ALDH1 may play a pivotal role in the emergence of malignant phenotypes, which would make it relevant for future direct targeted therapeutic approaches. These findings provide valuable insights for the study of LSCC, especially in the field of CSC markers. CD44 could be used as a potential prognostic value for the progression to oral malignancy, which results from the presence and the intensity of dysplasia in cancerous lesions. ^26^ As far as we know, the results presented here indicate a possible distinct pattern of CD44 in malignant and potentially malignant lesions, which leads to the hypothesis that characteristics such as alcohol consumption, in the presence of UV chronic exposition, could increase CD44 immunolabeling in slow-developing AC lesions; however, these factors could not maintain its expression in LSCC sites.

## Conclusion

In summary, although both ALDH1 and CD44 were associated with the presence of dysplasia in AC lesions, the present findings suggest that these CSC markers may be activated by different etiopathogenic pathways and predominate in distinct steps of oral carcinogenesis. CD44 may be more significantly related to the premalignant lesion, while ALDH1 can be predominantly linked to malignancy.
